# Fifty years of research in the *Scandinavian Journal of Work, Environment & Health*

**DOI:** 10.5271/sjweh.4135

**Published:** 2024-01-01

**Authors:** Alex Burdorf, Reiner Rugulies

**Affiliations:** 1 Department of Public Health, Erasmus Medical Center Rotterdam, Rotterdam, The Netherlands.; 2 National Research Centre for the Working Environment, Copenhagen, Denmark.; 3 Section for Epidemiology, Department of Public Health, University of Copenhagen, Denmark.

**Keywords:** history, research trend, trend

## Abstract

**Objective:**

The Scandinavian Journal of Work, Environment & Health was launched 50 years ago. In this paper we describe how research topics have changed over time.

**Methods:**

A complete list of all 2899 articles in the past 50 years was compiled. Each article was coded for type of exposure, type of health outcome, research design, first author, and country of correspondence address. Count of citations was based on the Scopus database.

**Results:**

Overall, the attention for chemical exposure in the first 30 years has shifted towards the psychosocial work environment, shift work, and physical work load. These shifts in exposure are mirrored by increased attention over time for mental disorders and musculoskeletal disorders. Cardiovascular disorders and cancer have been studied consistently over the past 50 years. Researchers from Scandinavian countries have been responsible for about 50% of the Journal’s content, while authorship has broadened to about 30 countries in recent years.

**Conclusion:**

During the past 50 years, some research topics have consistently remained highly visible in the Journal, whereas other topics have gained or lost interest. In terms of authors’ contribution, the Journal has its roots in research from the Nordic countries, but has evolved over time as a truly international periodical with a well-recognized position in research on occupational health.

Today, we enter the 50^th^ year of the *Scandinavian Journal of Work, Environment & Health*. The inaugural issue was published in January 1975 as a result of a merger between the *Swedish Nordisk Hygienisk Tidskrift* and the *Finnish Work-Environment-Health* periodical (1). National institutes in the Scandinavian countries supported the new journal and its aim to promote an international perspective on science in occupational health. At that time, Sven Hernberg from the Finnish Institute of Occupational Health was appointed as Editor-in-Chief, a position he held for 24 years. The last six years of his tenure, he was assisted by Mikko Härmä, who took over as Editor-in-Chief in 2000, supported by the appointment of Eira Viikari-Juntura as assistant Editor-in-Chief. Together, Mikko and Eira took the journal into the modern world of publishing, with an internet-based manuscript submission and editorial system complemented by online content. Gone were the days when authors mailed a thick envelop with a paper version with four copies to the editorial office, often receiving referee comments scribbled in the margin of the text. Today's Editors-in-Chief Alex Burdorf (appointed in 2018) and Reiner Rugulies (appointed in 2019) transitioned the Journal to open access (2).

The Journal’s first editions contained an eclectic collection of contributions, varying from animal studies and development of instruments for exposure studies in workplaces to observational epidemiology and reflections on quality and management of occupational health services. Gradually over the years, the Journal honed its emphasis on occupational epidemiology, placing stronger focus on observational studies investigating exposure–response relationships and experimental research on workplace-based interventions. In the past 25 years, we have stopped publishing animal studies, technical papers on ventilation at the workplace, and studies on environmental issues such as dietary intake of polychlorinated compounds through fish consumption.

## Becoming an international journal

**Figure 1:**
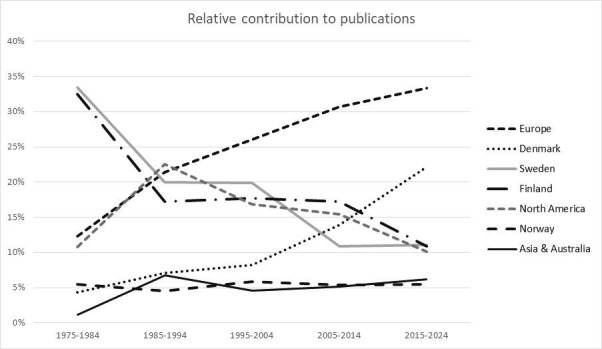
Relative contribution of countries to the scientific production in the *Scandinavian journal of Work, Environment & Health* over the past five decades.

Initially, the journal relied heavily on the prominent position of the national research institutes in Sweden and Finland, and their ties to academy in both countries. Figure 1 illustrates that, in the first decade, Swedish (33%) and Finnish (32%) researchers authored the vast majority of the more than 400 articles in the Journal. Their relative contributions dropped substantially, arguably linked to the closure of the Swedish National Institute for Working Life in 2007 and budget cuts in Finland in more recent years. Both Norway and Denmark had a share of about 5% in the first ten years, with strongly diverging trends thereafter. Nowadays, Danish researchers have a share of about 21%, the largest contribution of a single country to the scientific output of the Journal. Altogether, since 1985, researchers from Scandinavian countries have been responsible for about 50% of the Journal’s content.

Over the years, the authorship has broadened from 10 to about 30 countries in recent years. In the first 20 years, authors based in North America and United Kingdom contributed most to the growing international orientation of the Journal. In the past decade, The Netherlands became the country with the second most publications, responsible for about 50% of the share of content from Europe.

It is fair to conclude that the current *Scandinavian Journal of Work, Environment & Health* is still strongly rooted in its Nordic origins. But, with authors from over 30 countries, the Journal is certainly an international periodical with a well-recognized position in research on occupational health.

## Looking back into the future

In his reflection on 20 years of the *Scandinavian Journal of Work, Environment & Health*, Hernberg boldly stated that researchers should avoid “endless repetitions of previous work” (3), exactly 20 years before the international debate on research waste was launched (4). He was not afraid to give specific examples of over-researched topics, such as another study on how poor work postures contribute to shoulder pain, and studies showing that asbestos exposure causes mesothelioma in yet another country. His warning was in vain, as both topics have been published thereafter in our Journal repeatedly. In this celebratory 50^th^ year, we will have a contribution reflecting on the past, present, and future of research on musculoskeletal disorders, illustrating that research is still required for developing and evaluating targeted preventive strategies. Another contribution will focus on asbestos, using the differences in mesothelioma trends across countries to illustrate how reducing the use of asbestos and its eventual ban has been one of the most successful prevention strategies to reduce the work-related burden of disease.

In 1994, Hernberg also identified important knowledge gaps. Some suggestions have certainly been addressed in publications after 1994, such as shift work, biological monitoring, and exposure assessment strategies. The most cited paper in the period 2015–2023 was a systematic review on shift work and cardiovascular diseases (5). Likewise, Hernberg identified work ability, reproductive health, and mental disorders as important new research areas. The list of the ten highest cited papers in 2005–2014 contains two publications on work ability (6, 7). Mental disorders are now among the most prominent disorders in articles in our Journal.

It is interesting to mention also a few suggestions that have not been picked up by researchers seeking the *Scandinavian Journal of Work, Environment & Health* as their source of publication. The emerging interest in genetic susceptibility to specific exposures in the 1990s has resulted in tens of thousands of papers each year, often published in highly specialized biomedical or environmental health journals. Another topic on Hernberg’s list of priorities was the impact of unemployment on health and poor employment contracts. Only in recent years have we started to pay attention to precarious employment (8, 9) as well as unemployment and health (10).

As part of the 40 year celebration of the *Scandinavian Journal of Work, Environment & Health*, Härmä and Viikari-Juntura reflected on the world of scientific publishing (11). They discussed the amazing growth of open access journals, in terms of both the launch of many new journals and the enormous number of publications therein. It is no longer the question *whether* your paper will be published but *where.* In this avalanche of scientific production, high quality peer-review international journals are needed more than ever.

## Changing research agenda on exposure

The topics over the past 50 years of the *Scandinavian Journal of Work, Environment & Health* demonstrate clear trends in the research agenda. Table 1 shows the changes in distribution in exposure of interest over time. Overall, among the almost 2100 articles with an identifiable measure of exposure, 28% of all studies reported on a chemical exposure, most often organic solvents (180 studies) and pesticides (60 studies). Organic solvents were studied in relation to cancer and neurological, reproductive, and respiratory disorders. Pesticides were mainly studied for their associations with cancer and reproductive disorders. In the first 30 years, interest in chemical exposure topped the publication lists, but, in the past 10 years, <10% of all publications addressed a chemical exposure.

**Table 1:** Trends in relative rank of frequency of type of exposure in publications in the past 50 year, stratified by three periods.

Exposure of interest (% publications)	Rank 1975–1984	Rank 1995–2004	Rank 2015–2023
Chemicals (28%)	1	1	4
Psychosocial work environment (12%)	9	3	1
Shift work and working hours (11%)	8	6	2
Physical work load (10%)	4	2	3
Metals (6%)	2	4	7
Fibers (4%)	3	7	5
Handarm vibration (4%)	6	10	8
Biological agents (4%)	7	5	9
Dust (4%)	5	9	6
Electromagnetic fields (2%)	10	8	10

The largest change in exposure of interest relates to the psychosocial work environment. In the first 10 years, few papers mentioned psychosocial factors at work. In 1977, the first paper reported on a study among five fishermen in Norway, investigating heart rates and urinary catecholamine excretion rates in relation to the work schedule. The conclusion was that “contrary to general belief, bank fishing need not be unsuitable for older fishermen, provided an effective system of job rotation is practiced and the size of the crew is large enough to allow for an adequate amount of sleep even during periods of exceptionally good fishing” (12). The interest in the psychosocial work environment has grown steadily over the years and, in recent times, is the number one topic in our Journal. Since the early 1980s, most publications used the demand–control model (job strain model) to study how psychosocial working conditions influenced the occurrence of cardiovascular diseases. From the 1990s, the research areas rapidly expanded with other health outcomes, most notably mental disorders, and other psychosocial factors such as effort-reward imbalance, job insecurity, relational justice, leadership and sexual harassment (13).

Studies on shift work and working hours rose to the premier rank in the period 2005–2014 and are still ranked as the second most frequent topic in the past decade. This type of exposure has been linked to cardiovascular disorders, sleep disorders, injuries, mental disorders, and cancer. Already the first volume featured a study on irregular work hours and sleep duration among Swedish railroad workers (14). It is fascinating that, after all these years since this Swedish study and the aforementioned Norwegian study on fishermen (12), associations between shift systems and sleep disturbances are still high on the research agenda (15).

Over the years, some exposures of interest emerged quickly but also disappeared within a decade. During 1985–1994, more than 50 publications addressed exposure to hand-arm vibration, ranging from studies trying to establish the best exposure metrics and the optimal exposure assessment procedure to studies on clinical diagnosis of vibration-induced white fingers (in later years called hand-arm vibration syndrome) and exposure–response relationships. In the past 10 years, we have only published two papers on this type of exposure. This surely is not an indication that this exposure is no longer inducing health effects but that research interest has moved on to different areas. A similar trend is observed for studies on electromagnetic fields, which appeared regularly during the period 1985–2004, and thereafter rapidly evaporated.

## Changing research agenda on health

The changes in exposures of interest in research are also reflected in the health outcomes in articles published in the *Scandinavian Journal of Work, Environment & Health*. The strong shift towards the psychosocial environment is mirrored by an increased interest in mental disorders (see table 2). Early studies addressed associations between organic solvents and neurological problems. An exemplary study from 1975 described how multiple measurements of mandelic acid in urine were associated with reduced psychomotor performance and visual memory losses during subsequent clinical examinations among workers exposed to styrene in manufacturing polyester plastic products (16). In these early days of exposure assessment strategies, this study clearly demonstrated the importance of tackling exposure variation by conducting five measurements per worker and carefully designing medical tests. A case-referent study on organic solvents and psychiatric disorders made use of the excellent work disability registers in Finland and applied a crude exposure–response matrix (17). In more recent years, publications focused on a large variety of psychosocial factors in relation to mental disorders, such as depressive symptoms (18) and burn-out (19).

**Table 2:** Trends in relative rank of frequency of type of health outcome in publications in the past 50 years, stratified by three periods.

Health outcome of interest (% publications)	Rank 1975–1984	Rank 1995–2004	Rank 2015–2023
Cancer (18%)	1	2	4
Musculoskeletal disorders (16%)	7	1	2
Circulatory diseases (14%)	2	4	3
Respiratory diseases (11%)	3	3	8
Mental disorders (8%)	8	7	1
Reproductive disorders (7%)	6	5	7
Injuries (4%)	9	9	5
All-cause mortality (4%)	5	8	10
Neurological disorders (4%)	4	6	9
Sleep disorders (3%)	10	10	6

Although already addressed in the very first issue of our Journal, musculoskeletal disorders rapidly gained attention from 1984 onwards and is currently still high on the relative rank of type of health outcome in our publications. Initially the focus was largely on physical load and low-back pain, but since the keystone publication in 1993 on the role of psychosocial factors at work in occurrence of musculoskeletal disorders (20), research interest has broadened across exposure an health outcome of interest. Notwithstanding Hernberg’s critical remarks on needless research, there still are important knowledge gaps how physical load contributes to the onset and progression of various musculoskeletal disorders, as illustrated in recent publications linking job-exposure matrices to large study populations (21, 22).

Circulatory diseases, ie, primarily cardiovascular diseases, have always attracted interesting publications throughout the history of the Journal. Early papers were on heat stress and heart functioning in a climate chamber (23) and strenuous work postures and physiological response such as heart rate and blood pressure (24). This type of study, strongly routed in the work physiology tradition with laboratory experiments, has almost completely disappeared from our pages. Nowadays, epidemiological studies on cardiovascular diseases address a wide variety of risk factors, eg, welding fumes (25), psychosocial resources such as support and leadership (26), and night and shift work (27). There is now also attention for the intriguing question why leisure-time physical activity has beneficial effects for cardiovascular diseases, but occupational physical activity lacks this health enhancing impact (28).

Studies on cancer remain important for the Journal (18% of all publications). Cancer was most often studied in relation to chemical exposure (122 studies), most notably solvents, pesticides, and welding, asbestos (29 studies), metals (21 studies), and shift work (20 studies). From the early years, papers on asbestos and mesothelioma were regularly published, initially to determine exposure–response relationships for several cancer types in different industries. A well-received publication on diagnosis of asbestosis and attribution to asbestos became well-known as the Helsinki criteria for asbestosis, first published in 1997 and revised in 2015 (29). In more recent years, publications also described the impact of environmental asbestos exposure on the general population and the predicted burden of mesothelioma in the future (30). The studies on chemicals and cancer covered many different chemical agents, often taking advantage of the excellent cancer and mortality registers in the Nordic countries, where information on job titles can be linked to a job-exposure matrix for exposure characterization.

## Emerging evidence from interventions

Less than 6% of the Journal’s publications have been dedicated to the evaluation of the effectiveness of an intervention. Before 2000, hardly any intervention study had been published. One of the first ones from 1993 described that the introduction of short periods of bright light during night shifts among 15 young nurses did not seem to influence their tolerance to night work, as determined by melatonin excretion (31). Several studies on bright light interventions followed. Recently a comprehensive laboratory simulation study with randomization of 29 adults over four weeks showed promising results of introducing bright light regimes during night shifts on circadian adaptation (32).

In the first decade of the 21^st^ century, interventions on return-to-work became popular, partly due to targeted funding schemes in a few countries. A well-cited randomized controlled trial (RCT) in The Netherlands showed that the usual care approach actually outperformed the newly designed return-to-work intervention (33). An evaluation of a return-to-work intervention in three municipalities in Denmark showed strong differences across the intervention sites, illustrating the major importance of contextual factors (34).

In the past 15 years, health promotion programs, especially exercise and educational interventions, have become popular and are often conducted as an RCT. One of the best cited papers on this topic reported that duration and intensity of neck and shoulder symptoms was lower than in the reference group following two different specified worksite physical-activity interventions. The authors also pointed out that any effects on important measures for companies, such as sickness absence and work ability, could not be demonstrated due to the rather healthy participants (35).

Overall, the large majority of workplace interventions published in the Journal have been at the individual level, presumably because such interventions can be more easily randomized than other types (36). A recent scoping review of 80 systematic reviews on workplace mental health interventions showed beneficial effects of some individual-orientated interventions, but also concluded that there is a lack of evidence on organization and system level factors interventions (37). However, this conclusion is contrasted by a recent umbrella review of 52 systematic reviews, covering almost 1000 studies on organizational-level workplace interventions, that concluded that organizational-level interventions can be effective with regard to improving psychosocial working conditions and selected health outcomes, including burnout (38). These conflicting conclusions on the evidence for the effectiveness of organizational interventions likely reflect, among other things, disagreements about the most appropriate methods to evaluate workplace interventions. It is humbling to note that, almost 25 years ago, Griffiths (39) made a passionate plea to go beyond the experimental paradigm to evaluate organizational interventions.

In recent years, we have seen the introduction of methods for causal inference in observational studies, also known as natural experiments. The essence of such a study is that the assignment of individuals to intervention or control conditions is completely outside the control of the researcher or participants. A classic example is the introduction of a new nationwide policy whereby the timing of the introduction can be regarded as a truly random process. These methods allow evaluation on policies that affect worker’s health. For example, Finnish researchers showed that the use of part-time sick leave during the first three months of sickness absence resulted in earlier return-to-work among persons with mental disorders and musculoskeletal diseases (40). Likewise, the introduction of a work reintegration programme in the construction industry in Ontario, Canada, was associated with less disability days after a work-related injury (41).

## Most-cited papers

In the last 50 years, we have noted an extremely skewed distribution in citations of papers published in the Journal. Some papers, especially reviews, are very well cited whereas other barely received recognition by their peers. In table 3, we present the top cited papers in the history of the Journal, distinguishing reviews from original papers. The highly cited reviews reflect the choice in research topics that have featured in the Journal pages the most: psychosocial work environment, working hours, and musculoskeletal disorders. The list of the ten most-cited original articles is a bit more surprising. Three publications describe the development of a particular instrument (ie, a questionnaire) that is widely accepted by the research community. Several original contributions introduce appealing theoretical frameworks that are often used by researchers to design their own study. Other original contributions have demonstrated such convincing results that they have formed the building foundation for new research. Although this analysis is not intended to guide the editorial policy of the Journal – we are aware of the biases in citation practice (eg, excellent and important studies may get low citation numbers, if their results show null findings) – the table illustrates which contributions have found their scientific readership the most.

**Table 3:** Publications with the highest number of citations in the 50 year history

Author		Title	Year	Citations
**Reviews**				
	Stansfeld et al (42)	Psychosocial work environment and mental health - a meta-analytic review	2006	1220
	Bongers et al (20)	Psychosocial factors at work and musculoskeletal disease	1993	869
	Kivimäki et al (43)	Work stress in the etiology of coronary heart disease - a meta-analysis	2006	609
	Belkic et al (44)	Is job strain a major source of cardiovascular disease risk?	2004	501
	Bøggild et al (45)	Shift work, risk factors and cardiovascular disease	1999	474
	Burdorf at al (46)	Positive and negative evidence of risk factors for back disorders	1997	447
	Armstrong et al (47)	A conceptual model for work-related neck and upper limb musculoskeletal disorders	1993	446
	Van der Hulst (48)	Long workhours and health	2003	413
	Hoogendoorn et al (49)	Physical load during work and leisure time as risk factors for back pain	1999	406
	Bannai et al (50)	The association between long working hours and health: A systematic review of epidemiological evidence	2014	388
**Original articles**				
	Kristensen et al (51)	The Copenhagen Psychosocial Questionnaire - a tool for the assessment and improvement of the psychosocial work environment	2005	922
	Geurts et al (52)	Recovery as an explanatory mechanism in the relation between acute stress reactions and chronic health impairment	2006	569
	Elo et al (53)	Validity of a single-item measure of stress symptoms	2003	514
	Demerouti et al (54)	Burnout and engagement at work as a function of demands and control	2001	452
	Johnson et al (55)	Combined effects of job strain and social isolation on cardiovascular disease morbidity and mortality in a random sample of the Swedish male working population	1989	401
	Ahlstrom et al (7)	The work ability index and single-item question: associations with sick leave, symptoms, and health – A prospective study of women on long-term sick leave	2010	399
	Punnett et al (56)	Back disorders and nonneutral work trunk postures of automobile assembly workers	1991	391
	Viola et al (57)	Blue-enriched white light in the workplace improves self-reported alertness, performance and sleep quality	2008	341
	Theorell et al (58)	Changes in job strain in relation to changes in physiological state – a longitudinal study	1988	338
	Vartia et al (59)	Consequences of workplace bullying with respect to the well-being of its targets and the observers of bullying	2001	325

## Celebrating 50 years of science

In this 50^th^ volume of the *Scandinavian Journal of Work, Environment & Health*, each issue will contain one publication reflecting on key research topics in the past, present and future of occupational health research. As editors, we hope this series will stimulate future research and foster the Journal’s position as a premier platform for innovative knowledge on how we can contribute to a safer and healthier workplace all over the world.
